# Optimal gut-brain equilibrium: moderate physical activity, microbiome, and the hormetic pathway to cognitive wellbeing

**DOI:** 10.3389/fmicb.2026.1778491

**Published:** 2026-08-28

**Authors:** Huijun Du, Junfeng Zhang, Jin Xie, Xiaoyan Chen

**Affiliations:** 1 Hubei Provincial Clinical Research Center of Central Nervous System Repair and Functional Reconstruction, Taihe Hospital, Hubei University of Medicine, Shiyan, Hubei, China; 2 Department of Rehabilitation, Taihe Hospital, Hubei University of Medicine, Shiyan, Hubei, China; 3 Hubei University of Chinese Medicine, Wuhan, China; 4 School of Basic Medical Sciences, Hubei University of Chinese Medicine, Wuhan, China

**Keywords:** cognitive, gut-brain axis, microbial functional, microbiome, physical activity

## Introduction

The beneficial impact of regular physical activity on brain health is unequivocal. Nevertheless, the research persists in contending with a seemingly straightforward inquiry: what is the optimal quantity and intensity of exercise required to enhance cognitive function? The research conducted by Cintado et al. (2025) presents a significant advancement by correlating an inverted-U, or hormetic, dose-response with hippocampal neurogenesis and memory, alongside the coordinated, intensity-dependent alteration of the gut microbiota, and by transmitting these effects through fecal microbiota transplantation (FMT) in murine models. This approach links two fundamental aspects of brain health-exercise and the microbiota-gut-brain axis within a mechanistic framework that is both testable and possibly effective.

During a period when the public health directive is to prolong healthy cognitive years by scalable and equitable interventions (De Miguel et al., 2021; de Vos et al., 2022; Livingston et al., 2020), the assertion that “moderate” exercise fosters a pro-cognitive gut-brain environment, whereas excessive exercise may diminish such benefits, is both scientifically sound and pragmatically relevant. The authors' comprehensive dataset-encompassing behavior, adult hippocampal neurogenesis (AHN), vascular and glial characteristics, microbial community composition, and FMT offers a unique, cohesive perspective of this domain (Cintado et al., 2025).

## Study overview and key findings

Adult male C57BL/6J mice were assigned to inactive control conditions or to 6 weeks of moderate treadmill running (RUN; 40 min/day at 1,200 cm/min), extended-duration running (RUNtime; 60 min/day), or higher-velocity running (RUNvel; up to 1,800 cm/min) (Cintado et al., 2025). Under the testing conditions used, the RUN group showed stronger performance in novel object recognition and localization tasks. It also had higher numbers of SOX2+GFAP+ neural precursors and DCX+CLR+ immature neurons in the dentate gyrus. Locomotor activity and anxiety-like behavior did not differ materially between groups, which reduces, but does not eliminate, concern that generalized arousal accounted for the cognitive findings (Cintado et al., 2025).

The microbiome data were consistent with a regimen-sensitive response. Moderate running increased alpha diversity and shifted beta diversity; several genera were associated with the composite cognitive index. In sedentary recipients, FMT from RUN donors, compared with FMT from RUNtime donors, was accompanied by better memory performance and higher neurogenic cell counts (Cintado et al., 2025). These observations make the microbiota a plausible contributor to the phenotype, not merely a parallel marker of training exposure.

However, the study compares a limited set of protocols. It therefore identifies the most favorable condition among the regimens tested, rather than an optimal dose across all possible combinations of frequency, intensity, time, mode, progression, and recovery. Exercise dose is multidimensional. Frequency and weekly volume influence cumulative exposure; intensity changes acute autonomic, metabolic, and inflammatory demands; duration and modality affect the balance of mechanical and energetic stress; and recovery determines whether a given load is tolerated or accumulated (Garber et al., 2011; Levin et al., 2021). The phrase “optimal dose” should consequently be used only as shorthand for an experimental optimum within the specific population, treadmill model, duration, and outcomes studied.

## Positioning within the literature

The results correspond with convergent research indicating that physical activity enhances cognitive health across the lifespan. The Lancet Commission identifies physical inactivity as a modifiable risk factor for dementia, underscoring the need of attainable aerobic exercise for cognitive resilience (Livingston et al., 2020). While human randomized controlled trials exhibit variability in cognitive outcomes, mechanistic studies increasingly indicate multi-system mediators, including exercise-induced neuromodulatory proteins. Significantly, plasma from exercised mice enhances memory through the complement regulator clusterin, demonstrating a circulating factor axis that complements the microbiota (De Miguel et al., 2021).

This work progresses beyond previous findings that exercise alters microbial populations and their metabolic capabilities (de Vos et al., 2022; Scheiman et al., 2019). It demonstrates that the dosage of exercise influences not only “if” but also “how” microbiota alter, and that a moderate-intensity profile is both transmissible and pro-neurogenic. The absence of group-specific variations in fecal SCFAs does not negate a gut-brain link; SCFAs and other metabolites such as indoles and bile acid derivatives can signal both locally and systemically, display regional gradients, and be swiftly used, diminishing fecal disparities (de Vos et al., 2022; Dalile et al., 2019). Furthermore, the intensity-dependent increase of propionate degradation aligns with findings that some bacteria alter energy pathways under significant physiological stress, potentially affecting neuromodulatory equilibrium (Scheiman et al., 2019; Dalile et al., 2019).

The microglial characteristics observed with more severe regimens enhanced Iba1 signaling without widespread pro-inflammatory cytokine activation, align with priming rather than explicit neuroinflammation (Cintado et al., 2025). Considering that bacteria influence microglial development and tone (Erny et al., 2015), the data indicate a manageable, intensity-sensitive gut-immune-neurogenesis axis that supports the inverted-U relationship.

## A framework for interpreting exercise dose

A useful next step is to separate the external exercise prescription from the internal dose experienced by the host. The external dose can be described with familiar FITT components: frequency, intensity, time, and type. Total work, rate of progression, and the interval between sessions should be added because they determine the cumulative load. The internal dose includes physiological strain, sleep and nutritional status, baseline fitness, disease burden, medication use, and the ability to recover. Two individuals given the same treadmill speed may therefore experience different internal doses and may show different microbial and cognitive responses.

This framework helps explain why an inverted-U relationship is biologically plausible without implying a single numerical threshold. At low exposure, the stimulus may be insufficient to produce durable neural or microbial adaptation. At a tolerable intermediate exposure, repeated exercise may support vascular, metabolic, immune, and neurotrophic processes that favor learning and AHN. At a higher or poorly recovered exposure, benefits may plateau or be offset by fatigue, altered substrate availability, sleep disruption, or stress-related signaling. This is a hypothesis about the balance of adaptation and strain, not evidence that vigorous exercise is generally harmful. Dose-response curves may differ by outcome, sex, age, training history, and disease state.

The novel contribution of this opinion is therefore a testable conceptual distinction: the exercise condition associated with the best outcome in a single experiment is an experimental optimum, whereas a translational exercise recommendation requires a broader dose-response surface. Such a surface should incorporate frequency, session intensity, duration, total weekly load, and recovery, and it should be evaluated against both cognitive endpoints and biological measures. The microbiome may help explain why the surface varies among hosts, but it cannot yet be used as a stand-alone marker of a person's cognitive “sweet spot.”

This distinction also prevents a common interpretive error. An inverted-U curve observed between three selected groups is compatible with hormesis, but it does not define the shape, location, or reproducibility of the curve outside those groups. The curve might shift when the training period is longer, when session frequency changes, or when mice are allowed to choose their own running pattern. It may also be non-linear for one outcome and monotonic for another. For example, a training exposure that improves memory could have a different association with metabolic fitness, affect, immune function, or injury risk. A useful dose-response study should therefore report the prescription, achieved workload, and indicators of internal strain rather than treating treadmill speed as the full exposure.

The framework further distinguishes mediation from moderation. A mediator is a factor through which exercise contributes to an outcome; a moderator is a factor that changes the size or direction of the exercise response. A baseline microbial profile could be a moderator if it predicts who benefits from a program, even if the program does not change that profile. A microbial metabolite could be a mediator if exercise changes its production and a targeted intervention alters the cognitive response. These alternatives require different designs and should not be conflated. This distinction is especially relevant for personalized exercise because an association between baseline microbiota and response does not establish that changing the microbiota will improve the response.

## How might the microbiota contribute?

The FMT experiment strengthens causal interpretation, but its meaning requires precision. Transferring donor communities from the moderate-running group to sedentary recipients produced a phenotype consistent with the donor group's cognitive and neurogenic advantage. This supports the sufficiency or transmissibility of some component of the donor microbiota for influencing the recipient outcome under the FMT conditions used. It does not by itself prove that microbiota changes are necessary for the cognitive benefit of exercise, that they fully mediate the effect, or that any identified genus is the active agent. Exercise also acts through circulation, skeletal muscle-derived factors, vascular adaptation, and neural activity; for example, exercise plasma can influence memory through clusterin (De Miguel et al., 2021).

A mediation claim requires evidence that exercise changes a defined microbial feature, that this feature changes the relevant host pathway, and that blocking or replacing it alters the exercise-cognition relationship. Future studies could combine longitudinal sampling with antibiotic depletion or gnotobiotic models, defined microbial consortia, metabolite supplementation or depletion, and pathway-specific rescue experiments. These approaches should be designed carefully because antibiotics, germ-free status, and transplantation procedures can themselves alter immune development, metabolism, and behavior. The appropriate conclusion at present is that the microbiota is a candidate mediator and a demonstrably transferable contributor, rather than the sole pathway linking exercise to cognition.

Three causal claims should be kept separate. First, exercise may be associated with microbial differences. Second, a donor microbial community may be sufficient to alter a recipient phenotype in a controlled transfer experiment. Third, a specific microbial change may be necessary for exercise to improve cognition. The first claim can be supported by observational profiling, and the second is supported in part by the FMT result reported by Cintado et al. (2025) The third requires an intervention that disrupts or restores the proposed pathway while the exercise exposure is held constant. Full mediation is a stronger claim still: it would require showing that the exercise effect is substantially reduced when the microbial mechanism is blocked and restored when it is reinstated. None of these distinctions minimizes the FMT finding; they define the next experiments needed to build on it.

Interpretation of FMT also depends on the recipient context. Engraftment, colonization resistance, diet, housing, sex, and the recipient's baseline microbiota can influence which donor organisms persist and which microbial functions are expressed. A transplant transfers a community and its ecological potential, not a single isolated mechanism. If a donor-associated effect is reproduced, the effect could arise from multiple organisms, microbially transformed metabolites, bacteriophages, or host responses to altered community structure. Reporting engraftment and functional change in recipients is therefore as important as describing donor communities. This is one reason that defined communities and metabolite interventions are valuable complements to whole-community FMT.

Several routes could connect a training-sensitive microbiota to the brain. Microbial metabolites, including SCFAs, indole derivatives, and bile-acid-related signals, may affect epithelial integrity, immune tone, enteroendocrine signaling, or afferent vagal pathways (de Vos et al., 2022; Dalile et al., 2019; Cryan et al., 2019). Microbes can also shape peripheral immune activity and microglial maturation (Erny et al., 2015). In Cintado et al. (2025), fecal SCFA concentrations did not differ across groups, whereas functional inference suggested intensity-related differences in propionate degradation. This pattern is informative but not decisive: fecal concentrations are an imperfect proxy for production, intestinal utilization, portal exposure, or brain-relevant signaling. The proposed mechanism should thus remain a chain of candidate links rather than a completed causal pathway.

A restrained reading of the microglial findings is also warranted. Increased Iba1 signal in the more demanding regimens without broad pro-inflammatory cytokine activation may reflect altered microglial state, but Iba1 alone does not distinguish priming, activation, morphology, or functional consequence (Cintado et al., 2025). More detailed cell-state markers, regional analyses, cytokine and chemokine profiling, and temporal sampling are needed before concluding that a particular microbial pathway drives microglial changes. This uncertainty does not weaken the rationale for studying a gut-immune-brain route; it identifies the measurements needed to resolve it.

Diet and recovery should be considered part of the mechanism, not simply nuisance variables. Fermentable substrate availability can influence which microbial functions are expressed, while insufficient energy intake, dehydration, disrupted sleep, or repeated unaccustomed exertion can change intestinal physiology and immune signaling (de Vos et al., 2022; Dalile et al., 2019; Cryan et al., 2019). These factors may amplify, attenuate, or reverse the microbial response to an identical exercise prescription. Their relevance does not imply that dietary fiber or any one metabolite should be treated as a substitute for physical activity. It means that exercise-microbiota studies should measure and, where possible, standardize dietary intake, timing of meals and samples, hydration, and recovery indicators. This will make reported microbial differences more interpretable and reduce the risk of attributing a dietary or stress-related effect to exercise intensity alone.

## Methodological strengths and limitations

The study has notable strengths. It used predefined treadmill regimens without aversive stimuli, behavioral tasks that were sensitive to hippocampal function, and a coordinated assessment of cognition, AHN, vascular and glial measures, microbial composition, predicted microbial functions, fecal metabolites, and FMT (Cintado et al., 2025). The FMT arm is particularly valuable because it tests whether a donor-associated phenotype can be transferred rather than relying only on correlations between exercise and microbial abundance.

The limitations define the scope of the conclusion. Only male mice were studied, the microbiome and FMT samples were small, and forced treadmill running does not reproduce voluntary human activity. The comparison did not independently vary frequency, duration, intensity, or recovery; increasing duration and speed can alter total work and internal strain at the same time. In addition, 16S rRNA sequencing and predicted pathways cannot identify strain-level functions or directly quantify metabolites. Fecal samples provide a useful but incomplete window into cecal, portal, and systemic exposure. These limitations favor a cautious interpretation of both dose and mechanism.

The analytic strategy should match this scope. Correlations between individual genera and cognition can generate hypotheses, but they are vulnerable to compositional effects, multiple testing, and confounding by the overall intervention. Predicted functional pathways are similarly useful for prioritization but should not be treated as measured metabolism. Future reports can strengthen inference by prespecifying a small number of primary cognitive and microbial outcomes, reporting effect sizes and uncertainty intervals, accounting for cage or litter effects when relevant, and testing whether findings replicate in independent cohorts. Integrating microbial measurements with exercise physiology will be more informative than seeking a single “beneficial” genus.

## What is meant by gut-brain equilibrium?

In this context, equilibrium does not mean a fixed microbial composition or a single physiological state that should be reached by every person. The microbiota is dynamic, and healthy adaptation can include short-lived shifts after exercise, meals, sleep loss, illness, or travel. The term is used here to describe a functional balance in which the exercise stimulus is compatible with recovery and does not appear to undermine the cognitive and neurogenic outcomes being measured. A favorable balance may be achieved by different prescriptions in different hosts. It should therefore be assessed through convergent outcomes rather than inferred from the abundance of one organism or one metabolite at a single time point.

This functional definition has practical consequences for interpretation. A lower diversity measure after a single training block is not automatically harmful, and a higher diversity measure is not automatically beneficial. Likewise, a change in a taxon that is statistically associated with cognition does not identify a treatment target without evidence of function and causality. The most useful microbiome measures will be those that help connect a reproducible microbial function to a prespecified host pathway and meaningful cognitive outcome. Until that evidence is available, the language of equilibrium should remain descriptive and hypothesis-generating rather than diagnostic or prescriptive.

## Priorities for causal and translational research

The first priority is a genuine multidimensional dose-response design. Preclinical experiments should vary frequency, intensity, duration, total weekly volume, and recovery separately where feasible, and should include both sexes and voluntary-activity comparisons. Measurements should distinguish acute responses after a single session from chronic adaptations after repeated training. Repeated sampling will help determine whether microbial and metabolite shifts precede, accompany, or follow cognitive and neurogenic changes.

The second priority is mechanistic resolution. Shotgun metagenomics, metatranscriptomics, and targeted plus untargeted metabolomics can be combined with absolute microbial quantification. Samples from feces, cecal contents, and blood would clarify where a signal is generated and how it is distributed. Parallel measures of intestinal barrier function, immune mediators, vagal signaling where appropriate, neurotrophic factors, and microglial state could then be incorporated into prespecified mediation models. A mediation analysis is not a substitute for intervention, but it can identify which links are most suitable for experimental testing.

The third priority is translation without premature personalization. Human trials should compare exercise programs that are matched or transparently different in total work, use objective adherence and recovery measures, document diet and medication use, and prespecify cognitive outcomes. Stool and blood collection should be timed consistently relative to the last exercise session, because acute and habitual exercise may not have the same microbial signature. The research question is not whether one universal dose exists. It is whether a range of sustainable exercise exposures improves cognitive outcomes and whether microbial features explain who benefits, under which dietary and recovery conditions, and by which biological routes.

Factorial and adaptive designs would be especially informative. A factorial study could test moderate and higher exercise exposures against different dietary substrate conditions while holding energy intake and sample timing as constant as practical. An adaptive design could adjust session duration or intensity according to prespecified recovery markers and determine whether those adjustments preserve cognitive and microbial benefits. Such studies should avoid treating an exploratory microbial association as a clinical decision rule. Instead, their first goal should be to estimate response heterogeneity and identify reproducible candidate pathways. Only after replication and intervention-based validation should a microbial measurement be considered for individualization.

Human translation also requires endpoints that are feasible and meaningful. Cognitive testing should use validated measures selected for the expected domain and population, with attention to practice effects. Brain imaging, perfusion measures, or blood biomarkers may add value in well-resourced studies, but they are not direct equivalents of rodent AHN markers. Trials should be powered for sex-stratified and age-stratified analyses where possible, because the dose that is tolerable and effective may differ across life stage, health status, and prior activity. Transparent reporting of adverse events, fatigue, sleep, and discontinuation is essential when comparing higher exercise loads.

## Implications for practice and policy

Clinical implementation must emphasize sustainable, moderate-intensity aerobic prescriptions for cognitive health, defined by individualized targets like perceived exertion or percentage of heart rate reserve, and supplemented by regular monitoring for overexertion. This method adheres to public health recommendations, is practical in primary care and community environments, and promotes prevention-oriented techniques that can reduce inequities by prioritizing accessible, consistent activity over resource-heavy training models.

Future practices and policies ought to incorporate microbiome endpoints into lifestyle trials to elucidate mediation and identify responders, employing scalable technologies like as stool samples and fundamental metabolomics in conjunction with cognitive assessments and, when practical, neuroimaging. Costs can be minimized through tiered sampling at baseline, mid-intervention, and completion, as well as by standardizing analytic workflows and reporting. Governance frameworks are essential for microbiome-informed methods; while fecal microbiota transplantation is not a cognitive therapy, its mechanistic application highlights the necessity of standardized donor screening, biobanking, and safety management (Cammarota et al., 2019). Equitable access to secure exercise facilities and fiber-rich nutrition must be integrated into program design to guarantee that microbiome-mediated advantages are widely and justly disseminated.

## Real-world feasibility and equity

Moderate-intensity aerobic exercise is economical, adaptable, and secure when customized to individual capabilities (Bull et al., 2020). Integrating movement counseling into standard care, establishing secure public areas for walking or cycling, and pairing programs with nutritional support to address the “fiber gap” can improve microbiota responsiveness and diminish structural obstacles. Measurement pragmatics advocate for concise, verified cognitive assessments, optional hippocampus imaging where accessible, and cost-effective stool assays in research environments. The social benefits of maintaining cognitive function via decreased care requirements and improved wellbeing are significant and correspond with person-centered, community-focused health promotion.

A priority research trajectory should initiate with human dose-finding trials that directly contrast moderate and high-intensity aerobic training, employing predetermined cognitive objectives and validated indicators of adult hippocampal neurogenesis, including hippocampus volume and perfusion. These studies must include extensive microbiome and metabolome profiling, provide sufficient power for sex-stratified analysis, and thoroughly control for recovery, sleep, and nutrition via standardized fiber consumption to mitigate confounding factors. Mechanistic resolution must progress beyond 16S rRNA surveys to shotgun metagenomics, together with both targeted and untargeted metabolomics of potential mediators, merged with host multi-omics. Simultaneous examination of vagal and immunological pathways will be crucial to delineate afferent signaling pathways connecting gut microbial activity to neurogenic and cognitive effects.

To elucidate hormesis over time and facilitate translation, longitudinal designs must chart trajectories from initiation to consolidation and maintenance, examining whether shifts in microbial and microglial populations precede or succeed cognitive and neurogenic improvements, and whether training periodization or nutritional enhancement can restore benefits when intensity exceeds the optimal range. Simultaneously, the development of actionable biomarkers for personalization is essential: composite indices that amalgamate training load, heart rate variability, inflammatory tone, and microbial characteristics could assess proximity to the cognitive “sweet spot,” facilitating adaptive modifications in clinical and community programs. This paradigm would facilitate mechanism-informed, egalitarian implementation and expedite the identification of changeable mediators suitable for scalable treatments.

## Conclusions

Cintado et al. (2025) provide evidence that, among the treadmill conditions tested in male mice, a moderate regimen was associated with the strongest cognitive and AHN outcomes and with a donor microbiota capable of transferring part of that phenotype. The study is best interpreted as evidence for an experimental hormetic pattern and for a transferable microbial contribution. It does not establish a universal optimal exercise dose or complete microbial mediation of exercise-induced cognitive benefit.

The broader proposal is that exercise hormesis should be studied as an interaction among prescribed load, internal strain, recovery, host context, and microbial function. This framing makes clear predictions: altering a component of dose or recovery should change the biological response; defined microbial or metabolite interventions should alter only specific links in the pathway; and dose-response curves should vary across hosts. Testing these predictions will show whether the microbiota can become a useful explanatory and, eventually, actionable component of exercise programs for cognitive health.
